# Apparent Digestibility Coefficients of Seven Animal Protein Ingredients for Coho Salmon (*Oncorhynchus kisutch*) Post-Smolts

**DOI:** 10.1155/anu/6645799

**Published:** 2025-11-25

**Authors:** Hairui Yu, Xinyue Zhang, Leyong Yu, Jiayi Zhang, Rongyu Yan, Lingyao Li, Govindhrajan Sattanathan, Abdur Rahman

**Affiliations:** ^1^Key Laboratory of Biochemistry and Molecular Biology in Universities of Shandong (Weifang University), Weifang Key Laboratory of Coho Salmon Culturing Facility Engineering, Institute of Modern Facility Fisheries, College of Biology and Oceanography, Weifang University, Weifang 261061, China; ^2^College of Fisheries, Huazhong Agricultural University, Wuhan 430070, China; ^3^Department of Physiology and Pharmacology, Karolinska Institutet, Stockholm 17177, Sweden; ^4^Shandong Collaborative Innovation Center of Coho Salmon Health Culture Engineering Technology, Shandong Conqueren Marine Technology Co. Ltd., Weifang 261108, China; ^5^Weifang Key Laboratory of Salmon and Trout Health Culture, Conqueren Leading Fresh Science and Technology Inc. Ltd., Weifang 261205, China; ^6^Department of Animal Sciences, University of Veterinary and Animal Sciences, Lahore 54000, Pakistan

**Keywords:** animal protein ingredients, digestibility, energy, nutrients, *Oncorhynchus kisutch*

## Abstract

The present study determined the apparent digestibility coefficients (ADCs) of dry matter, nutrients, and energy in selected seven animal protein ingredients, including Peruvian fish meal (PFM), native fish meal (NFM), Antarctic krill meal (AKM), native shrimp meal (NSM), poultry by-product meal (PBPM), meat and bone meal (MBM), and hydrolyzed feather meal (HFM) for coho salmon (*Oncorhynchus kisutch*) post-smolts (initial body weight: 212.73 ± 5.15 g). Using yttrium oxide (Y_2_O_3_, 0.5 g/kg) as an inert marker, a reference diet was formulated to contain ~440 g/kg of crude protein and 200 g/kg of crude lipid, whereas the test diets were composed of the reference diet and one of the test ingredients at a ratio of 70:30. The dry matter ADCs of the test animal protein ingredients ranged from 65.14% to 83.77%. The ADC of energy followed a similar trend to that of dry matter (69.03%–87.52%). The crude protein and lipid in all the test ingredients were well digested by the post-smolts with ADCs ranging from 74.12% to 91.67% and 75.44% to 92.38%, respectively. The MBM and HFM had significantly (*p* < 0.05) lower amino acid ADCs than other animal protein ingredients, resulting in lower levels of protein digestion in these two terrestrial animal ingredients. The ADCs of phosphorus varied greatly among the animal protein ingredients, with the highest in PBPM (70.52%) and the significantly (*p* < 0.05) lowest in MBM (37.31%). These ADCs data suggest that PFM, NFM, AKM, NSM, and PBPM can be prioritized as high-quality protein sources and provide more accurate information for the nutrient and energy utilization of coho salmon.

## 1. Introduction

The global output of farmed products reached 130.9 million tons in 2022, exceeding capture fisheries for the first time, and the two sectors jointly contributed about 15% of the global supply of animal protein [1]. Modern intensive aquaculture production relies on the use of aquafeed, which is the largest single source of cost, and determines the efficiency and benefit of farming operations [2]. As a high-quality but expensive protein ingredient, Fish meal is typically used at a rate of 30%–60% in commercial diets for carnivorous aquatic species [3, 4]. However, the increasing demand for fish meal in aquaculture, coupled with the depletion of marine fisheries resources, has resulted in high cost and unstable supply of fish meal [5, 6]. Therefore, alternative proteins are necessary to be found to replace fish meal [7, 8]. Many studies have aimed to overcome the “feeding fish to fish” paradox by substituting fish meal with other protein sources [9]. Except fish meal and shrimp meal, some terrestrial animal protein ingredients including poultry by-product meal (PBPM), meat and bone meal (MBM), and hydrolyzed feather meal (HFM) are affordable protein sources with lower level of antinutritional factors and higher level of essential amino acids (EAAs) than plant protein ingredients, and thus are widely used in aquafeeds [10–19].

Fish obtain the necessary nutrients by ingesting feed, which must undergo a series of digestive processes to break down large molecules of organic matter into simple, small molecules before they can be absorbed. The primary factor causing the variation in the nutritive value of feedstuffs is the loss in the form of indigestible matter in the feces. The apparent digestibility coefficients (ADCs) provide valuable indicators of energy and nutrient availabilities, and thus providing a reasonable basis for formulating diets to meet specific nutrient levels [20, 21]. Furthermore, the selection of ingredients with high ADCs can reduce the waste of nutrients and decrease the discharge of undigested components into surrounding water [22], which helps maintain water quality, reduce environmental pollution, and support the sustainability of aquaculture [23].

Coho salmon (*Oncorhynchus kisutch*) is mainly inhabited the coastal area of the northern Pacific Ocean [24]. Coho salmon have a moderate fat content, delicious flesh, and are rich in highly unsaturated fatty acids, which can promote the development of the brain and vision, and prevent cardiovascular and cerebrovascular, and other diseases, and are therefore highly favored by consumers [25, 26, 27]. The rapid development of coho salmon farming has made the development of feed particularly urgent. However, there are still lacking reports on the feed ingredient ADCs data for this cold-water carnivorous species. Therefore, the ADCs of selected seven animal protein ingredients commonly used in commercial feed, including Peruvian fish meal (PFM), native fish meal (NFM), Antarctic krill meal (AKM), native shrimp meal (NSM), PBPM, MBM, and HFM, were determined in order to provide a basis for the development of highly efficient and environmentally friendly feed for coho salmon.

## 2. Materials and Methods

### 2.1. Diets Preparation

The animal protein ingredients tested for ADCs included PFM, NFM, AKM, NSM, PBPM, MBM, and HFM. Yttrium oxide (Y_2_O_3_, 0.5 g/kg) was used as an inert marker. The reference diet contained ~440 g/kg of crude protein and 200 g/kg of crude lipid, whereas the test diets were composed of the reference diet (Table 1) with one of the test ingredients at a ratio of 70:30 according to the method of Cho and Slinger [28]. The chemical composition of the tested ingredients and diets is shown in Tables 2 and 3, respectively. Each test ingredient was ground through a 150 µm sieve. All the experimental diets were extruded into pellets (3.3 mm in diameter × 4.0 mm length), and then air-dried and stored at −20°C until fed.

### 2.2. Rearing Trial

The feeding trial was performed in strict accordance with the Guide for the Experimental Animal Management Methods at Weifang University (Approval Number 202203056). The coho salmon were provided and raised in a Joint Salmonidae Health Culture Research Center (Weifang, China). Before the experimental grouping, all the post-smolts were cultured in an indoor earthen pond (30.0 m length × 6.0 m width × 2.5 m depth) and fed the reference diet for 3 weeks. Thereafter, the post-smolts were fasted for 24 h, and 100 individuals (body weight: 212.73 ± 5.15 g) were assigned into each of the 24 circular culturing tanks (6.0 m in diameter and 2.5 m in depth) with three tanks per diet. The post-smolts were fed to apparent satiation three times daily (7:20, 12:20, 17:20) under a natural day-to-night cycle for 10 weeks. The water (temperature, 14.9 ± 0.7°C; dissolved oxygen, 9.5 ± 0.4 mg/L; pH, 7.1 ± 0.2) supplied in this experiment was filtered underground cold water.

### 2.3. Feces Collection

After adapting to the 2-week rearing conditions, the fecal samples were collected from all the post-smolts in each tank 5 h after the feeding (7:20–12:20) according to the method of Austreng [29]. The post-smolts were anesthetized with 30 mg/L tricaine methanesulfonate (MS-222) before sampling, and the feces were stripped every 7 days until sufficient feces were collected. The pooled fecal samples from each tank were stored at −20°C for the subsequent analysis.

### 2.4. Analytical and Calculating Methods

The nutrient and yttrium contents of the diets and feces samples were analyzed according to AOAC [30]. In briefly, the samples were dried to a constant weight at 105°C to determine the moisture. The crude protein (nitrogen × 6.25) was determined by the Kjeldahl apparatus (Kjeltec 8400, FOSS Analytical Co., Ltd., Suzhou, China). The crude lipid was extracted using petroleum ether by the Soxhlet apparatus (ST243 Soxtec, FOSS Analytical Co., Ltd., Suzhou, China). The ash was evaluated by incinerating the samples in a Muffle furnace at 550°C for 24 h. The phosphorus and yttrium were determined using inductivity-coupled plasma mass spectrometry (ICP-OES Optima 5300DV, Perkin Elmer Corporation). The gross energy was determined using an adiabatic bomb calorimeter (Parr Instrument Company, Moline, USA). The amino acids were measured using an automatic analyzer (Model A300, MembraPure GmbH, Frankfurt, Germany).

The ADC of dry matter, nutrients, and energy in the diet was calculated accorded to Cho and Kaushik [31]:

ADC of dry matter (%) = 100 − (% yttrium in feed)/(% yttrium in feces) × 100.

ADC of nutrient or energy (%) = 100 − (% yttrium in feed)/(% yttrium in feces) × (% nutrient or energy in feces)/(% nutrient or energy in feed) × 100.

The ADC in the test ingredient was calculated according to Cho et al. [32]:

ADC of test ingredient (%) = 100/30 × (ADC in test diet – 0.7 × ADC in reference diet) × 100.

### 2.5. Statistical Analyses

The data were displayed as mean with standard deviation (mean ± SD, *n* = 3). The SPSS 25.0 software was applied for all statistical analyses. Data were analyzed by one-way analysis of variance (ANOVA), after verifying normality and homogeneity of variance, ascertaining the significant differences among the various treatments by Duncan's test. The statistical significance was at a 5% level.

## 3. Results

The dry matter ADCs of the tested animal protein ingredients ranged from 65.14% to 83.77% (Table 4), which were not significantly different (*p* > 0.05) among PFM, NFM, PBPM, and AKM, but significantly (*p* < 0.05) higher than those in MBM and HFM. The NSM had intermediate dry matter ADC and did not differ significantly (*p* > 0.05) from the other animal protein ingredients. The ADCs of crude protein and crude lipid were ranged at 74.12%–91.67% and 75.44%–92.38%, respectively. The protein ADCs of PFM, NFM, and AKM were significantly (*p* < 0.05) higher than those of MBM and HFM, while the protein ADCs of NSM and PBPM were intermediate and did not differ significantly (*p* > 0.05) from other animal protein ingredients. The lipid ADCs of PBPM and MBM were significantly (*p* < 0.05) lower than those of the other animal ingredients.

The MBM and HFM exhibited significantly (*p* < 0.05) lower amino acid ADCs than other animal protein ingredients (Table 4). The ADCs of EAAs were all above 85% in PFM, NFM, AKM, NSM, and PBPM, while those in HFM and MBM were between 73.35% and 80.69%. Similar trends were found in the ADCs of non-EAAs (NEAAs). The NEAA ADCs of PFM, NFM, AKM, NSM, and PBPM were ranged from 85.25% to 93.68%, while those in HFM and MBM were between 72.37% and 76.27%.

The phosphorus ADCs of the test ingredients varied greatly, ranging from 37.31% to 70.52%. The highest and lowest phosphorus ADCs were observed for PBPM (70.52%) and MBM (37.31%), respectively. The ADC of phosphorus in AKM (67.07%) was lower than that in PBPM but higher than that in PFM (63.81%), and significantly (*p* < 0.05) higher than that in NSM (61.23%), NFM (60.41%), and HFM (57.14%).

The gross energy ADCs were ranged from 69.03% to 87.52%, with the highest energy ADC for PFM and the lowest for HFM. The ADC of gross energy in MBM (72.62%) did not differ significantly (*p* > 0.05) from that in HFM and NSM (80.56%) but was significantly (*p* < 0.05) lower than that in PFM, AKM (86.26%), PBPM (85.19%), and NFM (83.04%).

## 4. Discussion

Proteins are the most abundant organic matter in fish tissues, accounting for about 70% of the dry matter [33] and contributing to growth and physiological functions. The amino acid composition and protein ADC of feed ingredients largely reflect their protein qualities. In this study, the coho salmon digested the fish meal and AKM well, which was consistent with the results observed in Atlantic cod (*Gadus morhua*), where AKM had a higher protein ADC (96.3%) than fish meal (93.3%), indicating that AKM can be used as a high-quality protein source in aquafeed [34]. The ADCs of protein from MBM (78.84%) and HFM (74.12%) by coho salmon were lower than those of other animal ingredients. Moutinho et al. [35] found that substituting fish meal with MBM significantly decreased the protein efficiency ratio of gilthead seabream (*Sparus aurata*), which was due to the lower digestibility and metabolic utilization of protein in MBM. The gilthead seabream and turbot (*Psetta maxima*) exhibited consistently higher crude protein digestibility of PBPM than that of HFM [36]. Another study on European seabass (*Dicentrarchus labrax*) showed that replacing 76% of fish meal with HFM significantly decreased the protein ADC [17]. However, the protein ADC of HFM for channel catfish (*Ictalurus punctatus*) was 89.83% [37], which was higher than the results obtained in coho salmon. Safari et al. [38] compared the effects of hydrolysate methods on feather meal and found that the broilers fed the diet with fermented HFM exhibited the highest protein digestibility, feed utilization, and growth performance. These variations may be due to the different fish species and assessment methods, and component variation of HFM caused by the different extent of keratin and/or processing techniques. The amino acid ADCs of PFM, NFM, AKM, NSM, and PBPM were significantly higher than those of MBM and HFM, which were in line with the protein digestibility. The ADCs of EAAs in HFM exceeded 73%; however, the HFM could not provide sufficient methionine, lysine, and histidine for the growth of coho salmon due to the imbalance of the amino acid profile and relatively low amino acid levels. The imbalance in the amino acid composition of HFM may be related to the loss of certain specific amino acids in feathers during the hydrothermal process, for example, methionine is a thermolabile amino acid that is easily oxidized at high temperatures [39]. Lysine is the first limiting amino acid in fish, and insufficient lysine in the diet may limit protein synthesis, accumulation, and growth of marine organisms [40]. The ADC of lysine in MBM for coho salmon was 76.92%, higher than the 63.17% reported by Mo et al. [41] for mandarin fish (*Siniperca chuatsi*), but lower than the 85.9% reported by Zhang et al. [42] for bullfrog (*Rana Lithobates catesbeiana*). These differences may be resulted by the different nutritional composition of MBM and variations in the cultured species. Moreover, Tazikeh et al. [43] found that excessive addition of MBM (50%) to the diet could impair the growth of whiteleg shrimp (*Litopenaeus vannamei*), while appropriate addition (25%) has no significant effect on the shrimp's growth. Therefore, the addition of MBM in the diet for marine organisms should be controlled to ensure good digestibility.

The dry matter ADC represents the overall level of nutrient digestion [44]. The dry matter ADCs of PFM, NFM, AKM, NSM, and PBPM (77.71%–83.77%) were significantly higher than those in MBM and HFM (65.14%–67.13%), indicating that the nutrients from the former five animal protein ingredients could be better digested and absorbed by coho salmon. Similar results were also observed in the studies on tilapia (*Oreochromis mossambicus*) [45], mulloway (*Argyrosomus japonicus*) [46], and red Seabream (*Pagrus major*) [47]. In contrast, Wang et al. [48] reported that for Japanese seabass (*Lateolabrax japonicus*), the dry matter ADC of MBM (68.71%) was significantly lower than that of fish meal (83.14%), whereas HFM (84.25%) and fish meal did not differ significantly in the dry matter ADC. The diet type and composition are important factors affecting fish energy acquisition. In this study, the trend of the gross energy ADC was similar to that of dry matter. In commercial feed for coho salmon, the dietary energy is mainly furnished by proteins and lipids, and the amount of digestible carbohydrates is low (<11%) [49]. The ADC of gross energy in coho salmon (69.03%–87.52%) was lower than that in tilapia (83.9%–94.0%) [50] and rockfish (*Sebastes schlegeli*) (73%–99%) [51]. The high ash content in feed may reduce the ADC of dry matter and energy, and the increase in ash content in MBM could be the reason for the decrease in ADC. Booth et al. [46] found that the energy ADC of feather meal was only 61% for Mulloway, which is similar to the result (69.03%) for coho salmon. HFM contains a large amount of keratin, which cannot be well digested by fish, obstructing the catabolism of nutrients and energy conversion, and is not conducive to the digestion and utilization of carnivorous fish that mainly rely on protein as the energy source. Although both the PBPM and MBM had the highest crude lipid content among the test ingredients, the lipid ADCs of PBPM and MBM were significantly lower than that of the other test ingredients. Ma et al. [52] found that compared to fish meal, the PBPM and MBM led to a lower lipid digestion rate in vitro and decreased feed utilization in golden pompano (*Trachinotus ovatus*). The coho salmon had relatively high ADCs of lipid in both AKM (92.38%) and fish meal (NFM and PFM, 91.13%–91.69%). Similarly, Hansen et al. [53] found that the Atlantic salmon (*Salmo salar*) had high lipid ADCs of AKM (97.1%) and fish meal (93.2%). Additionally, Wei et al. [54] found that the intake of AKM brought higher levels of n-3 polyunsaturated fatty acids in large yellow croaker (*Larimichthys crocea*). These results indicated that the lipids in AKM were more easily digestible, utilizable, and provided health benefits to fish compared to those in the MBM and PBPM.

Phosphorus is an essential mineral component for the growth of fish bones and scales, and other vital physiological functions. Phosphorus deficiency is an important cause of vertebra deformities in salmon [55]. The phosphorus ADC revealed that the more digestible phosphorus in PBPM and AKM than that in fish meal, while a high amount of phosphorus in MBM was hardly absorbed. This large variation might be caused by the different levels of bone phosphorus in the ingredients and the chemical form of phosphorus in the diets [56]. Although MBM had a high phosphorus content, it is mainly in the form of insoluble calcium phosphate, making it difficult for fish to absorb and utilize [57]. Zhou et al. [58] observed a similar result that the phosphorus ADC of MBM was significantly lower than that of PFM, even though the phosphorus content of MBM was 1.4 times that of PFM. HFM might lead to insufficient intake of phosphorus and cause skeletal diseases in salmon, making it unsuitable as a substitute for fish meal, and similar results have been found in catfish [59]. In comparison, AKM and PBPM can be preferably used as specific feed ingredients for salmon bone growth due to their high content of absorbable phosphorus.

Many studies have shown that completely or partially substituting fish meal with animal protein ingredients has no significant effect on the growth of fish [60]. Based on the analysis of the ADC results, AKM and NSM are suitable ingredients to replace fish meal and can provide adequate nutrition for the growth of coho salmon. European sea bass fed with AKM instead of fish meal had higher protein and lipid efficiency rates and lower cholesterol levels [61], and similar results were found in rainbow trout (*Oncorkynokus mykiss*) [62]. For the terrestrial animal protein ingredients, PBPM has been demonstrated as a potential fish meal substitute in specific fish species such as catfish, Nile tilapia (*O. aureus*), and largemouth bass (*Micropterus salmoides*) due to its high anti-inflammatory and antioxidant effects [63, 64, 65]. One of our studies showed that PBPM could replace 16.63%–17.50% of fish meal protein in the diet for coho salmon post-smolts [66]. Both MBM and HFM had relatively low ADCs of dry matter, protein, and amino acid, and the lowest ADCs of phosphorus and energy, which could be partially substituted for fish meal from the perspective of feed cost reduction.

Many factors can affect the accuracy of the apparent digestibility test results in aquatic animals, such as the method of digestibility measurement, the type of indicator, and the method of feces collection [67]. Y_2_O_3_ and chromium trioxide (Cr_2_O_3_) are the most commonly used exogenous indicators in digestibility determination trials [68, 69]. Shiau and Liang [70] found that supplementation of Cr_2_O_3_ decreased the protein efficiency ratio and protein deposition in tilapia (*O. niloticus* × *O. aureus*), thus affecting the nutrient digestibility and growth performance. It has also been demonstrated that Cr_2_O_3_ influenced carbohydrate metabolism to some extent [71]. Y_2_O_3_ has been used to determine the apparent digestibility in Atlantic salmon and rainbow trout [72, 73]. No report to date has investigated the negative impact of Y_2_O_3_ on protein digestion in fish. Therefore, Y_2_O_3_ was used as an exogenous indicator in the present study.

## 5. Conclusion

In summary, PFM, NFM, AKM, NSM, and PBPM can be selected as the preferred high-quality animal protein ingredients in coho salmon diets because of their higher ADCs than MBM and HFM. Although MBM and HFM have lower ADCs, their relatively lower costs make them suitable as a partial substitute for fish meal to reduce the feed cost. Further research is essential to evaluate the effects of these animal protein ingredients in replacing fish meal to determine their appropriate inclusion levels in the feed.

## Figures and Tables

**Table 1:** Reference diet formulation for determination of apparent digestibility coefficients in coho salmon (*Oncorhynchus kisutch*) post-smolts.

Ingredients	Concentration (g/kg dry matter)
Fish steak meal	300.0
Soy protein concentrate	250.0
Peanut meal	100.0
Beer yeast	30.0
High-gluten wheat flour	120.5
α–Starch	25.0
Fish oil	75.0
Soybean oil	75.0
Dicalcium phosphate	10.0
Vitamin premix^a^	5.0
Mineral premix^b^	5.0
Choline chloride	3.0
Ascorbic acid phosphate (350 g/kg)	1.0
Yttrium oxide (Y_2_O_3_)	0.5

*Note*: All the ingredients were provided by Shandong Conqueren Marine Technology Co., Ltd., Weifang, China.

^a^Composition (IU or g/kg vitamin premix): retinal palmitate, 10,000 IU; cholecalciferol, 4000 IU; α-tocopherol, 75.0 IU; menadione, 22.0 g; thiamin-HCl, 40.0 g; riboflavin, 30.0 g; D-calcium pantothenate, 150.0 g; pyridoxine-HCl, 20.0 g; meso-inositol, 500.0 g; D-biotin, 1.0 g; folic acid, 15.0 g; niacin, 300.0 g; cyanocobalamin, 0.3 g.

^b^Composition (g/kg mineral premix): AlK(SO_4_)_2_·12H_2_O, 123.7; CuSO_4_·5H_2_O, 32.0; CoCl_2_·6H_2_O, 49.0; FeSO_4_·7H_2_O, 707.0; MgSO_4_·7H_2_O, 4317.0; MnSO_4_·4H_2_O, 31.0; KI, 5.3; NaCl, 4934.0; Na_2_SeO_3_•H_2_O, 3.4; ZnSO_4_·7H_2_O, 177.0.

**Table 2:** Proximate composition and amino acid profile (g/kg) of the test ingredients.

Ingredients	Test ingredients						
	PFM	NFM	AKM	NSM	PBPM	MBM	HFM
Proximate analysis (g/kg dry matter)							
Dry matter	917.7	897.5	910.1	897.4	946.3	918.9	941.0
Crude protein	708.4	683.9	651.9	593.0	679.5	563.7	881.0
Crude lipid	117.4	123.3	69.8	90.9	130.3	136.6	24.5
Ash	190.8	199.7	139.7	172.8	136.3	268.8	25.8
Phosphorus	21.9	26.3	15.1	15.5	16.4	45.6	6.1
Gross energy (kJ/g)	21.6	21.5	19.1	18.9	21.7	19.8	22.9
Essential amino acids profile (g/kg in protein)							
Arg	66.5	65.8	60.0	55.8	55.7	69.5	61.2
His	24.8	17.8	19.7	19.2	14.2	16.0	6.0
Ile	46.9	41.1	48.2	50.5	23.3	33.8	48.6
Leu	71.7	70.9	70.6	76.5	47.6	57.1	82.1
Lys	84.6	73.6	72.1	74.4	40.7	50.2	18.5
Met	32.8	28.0	21.2	27.2	12.9	18.3	6.9
Phe	50.6	43.7	48.4	45.3	27.5	36.5	44.3
Thr	50.0	45.5	41.1	47.2	29.4	32.8	40.7
Val	49.7	48.9	46.7	53.6	31.4	48.3	58.7
Nonessential amino acids profile (g/kg in protein)							
Cys	9.1	7.7	15.0	9.8	2.5	2.3	33.4
Tyr	41.7	39.9	37.9	42.7	19.4	26.4	24.2
Ala	59.5	63.9	54.1	62.4	63.5	68.9	46.1
Asp	96.3	89.9	112.1	103.2	78.7	50.6	57.1
Glu	154.6	147.1	141.6	151.1	107.8	121.8	99.5
Gly	61.1	57.5	49.9	63.5	119.7	124.5	65.7
Pro	48.5	61.1	38.3	51.1	74.0	108.3	207.1
Ser	43.4	42.0	39.3	42.5	33.1	40.3	56.7

*Note*: Tryptophan could not be analyzed because of its destruction during acid hydrolysis.

Abbreviations: AKM, Antarctic krill meal; HFM, hydrolyzed feather meal; MBM, meat and bone meal; NFM, native fish meal; NSM, native shrimp meal; PBPM, poultry by-product meal; PFM, Peruvian fish meal.

**Table 3:** Proximate composition and amino acid profile of the reference and test diets.

Items	Reference diet	Test diets (a 70:30 mixture of the reference diet to test ingredient)						
		PFM	NFM	AKM	NSM	PBPM	MBM	HFM
Proximate analysis (g/kg dry matter)								
Dry matter	903.1	910.2	915.0	908.3	917.7	901.9	906.5	912.3
Crude protein	442.2	522.1	514.7	505.1	487.5	513.4	478.7	573.8
Crude lipid	200.3	175.4	177.2	161.1	167.5	179.3	181.2	147.6
Ash	106.9	132.1	134.7	116.7	126.7	115.7	155.5	82.6
Phosphorus	14.2	16.5	17.8	14.5	14.6	14.9	23.6	11.8
Gross energy (kJ/g)	22.4	22.1	22.1	21.4	21.3	22.2	21.6	22.5
Essential amino acids composition (g/kg in protein)								
Arg	71.6	69.5	69.3	67.1	65.8	65.3	70.9	66.8
His	26.9	26.0	23.2	24.1	24.1	21.8	23.0	17.3
Ile	44.4	45.4	43.1	45.9	46.6	36.0	40.6	46.3
Leu	76.0	74.2	74.0	73.9	76.2	64.7	69.3	78.8
Lys	60.0	70.0	65.4	64.7	65.3	52.4	56.5	40.9
Met	16.9	23.4	21.4	18.6	20.7	15.3	17.4	12.3
Phe	50.5	50.5	47.8	49.7	48.6	41.4	45.5	47.6
Thr	40.1	44.1	42.2	40.5	42.7	35.8	37.5	40.4
Val	46.9	48.0	47.7	46.8	49.3	40.7	47.4	52.3
Nonessential amino acids composition (g/kg in protein)								
Cys	9.7	9.4	8.9	11.8	9.7	6.8	7.1	20.6
Tyr	36.8	38.8	38.0	37.2	38.9	29.9	33.1	31.0
Ala	51.7	54.9	56.5	52.6	55.6	56.3	57.7	49.1
Asp	94.8	95.4	92.9	101.5	97.8	88.4	79.2	77.4
Glu	180.0	169.7	166.9	165.1	169.4	151.3	159.4	142.9
Gly	43.4	50.6	49.0	45.9	50.8	73.7	72.1	53.7
Pro	52.1	50.6	55.7	46.7	51.7	60.8	71.9	123.5
Ser	45.6	44.7	44.2	43.2	44.5	40.7	43.8	50.7

*Note*: Tryptophan could not be analyzed because of its destruction during acid hydrolysis.

Abbreviations: AKM, Antarctic krill meal; HFM, hydrolyzed feather meal; MBM, meat and bone meal; NFM, native fish meal; NSM, native shrimp meal; PBPM, poultry by-product meal; PFM, Peruvian fish meal.

**Table 4:** Apparent digestibility coefficients (ADCs) of dry matter, nutrients, and energy in the test ingredients for coho salmon (*Oncorhynchus kisutch*) post-smolts.

ADC (%)	Test ingredients						
	PFM	NFM	AKM	NSM	PBPM	MBM	HFM
Dry matter	83.77 ± 1.95^a^	81.42 ± 2.09^a^	79.21 ± 3.47^a^	77.71 ± 3.10^ab^	80.27 ± 3.30^a^	65.15 ± 3.14^b^	67.13 ± 2.89^b^
Crude protein	91.67 ± 1.75^a^	89.14 ± 2.39^a^	88.90 ± 2.27^a^	85.01 ± 3.43^ab^	87.92 ± 2.68^ab^	78.84 ± 1.82^b^	74.12 ± 2.61^b^
Crude lipid	91.68 ± 1.67^a^	91.12 ± 3.55^a^	92.38 ± 2.35^a^	88.19 ± 3.32^a^	76.95 ± 1.75^b^	75.44 ± 2.75^b^	87.18 ± 2.20^a^
Phosphorus	64.01 ± 2.39^ab^	60.41 ± 2.56^b^	67.07 ± 2.27^a^	61.23 ± 1.98^b^	70.52 ± 2.51^a^	37.31 ± 3.42^c^	57.14 ± 3.29^b^
Gross energy	87.52 ± 2.92^a^	83.04 ± 3.05^a^	86.26 ± 3.68^a^	80.56 ± 2.33^ab^	85.19 ± 2.76^a^	72.62 ± 3.76^b^	69.03 ± 3.32^b^
Essential amino acids							
Arg	95.21 ± 1.81^a^	92.73 ± 1.31^a^	90.87 ± 1.63^a^	89.14 ± 1.45^a^	91.78 ± 0.88^a^	80.69 ± 1.51^b^	76.35 ± 1.33^b^
His	90.16 ± 1.05^a^	89.58 ± 1.39^a^	90.77 ± 2.50^a^	86.11 ± 2.00^a^	87.59 ± 1.45^a^	78.19 ± 2.94^b^	77.07 ± 2.27^b^
Ile	93.92 ± 1.13^a^	88.66 ± 2.02^a^	90.96 ± 1.83^a^	87.48 ± 2.15^a^	87.50 ± 2.19^a^	77.04 ± 2.67^b^	75.55 ± 1.52^b^
Leu	91.52 ± 2.33^a^	88.03 ± 1.97^a^	89.11 ± 2.07^a^	85.37 ± 1.64^a^	88.49 ± 2.54^a^	77.80 ± 2.40^b^	77.01 ± 1.36^b^
Lys	92.39 ± 2.22^a^	87.56 ± 2.28^a^	87.43 ± 1.34^a^	86.29 ± 1.85^a^	89.55 ± 1.26^a^	76.93 ± 2.29^b^	77.88 ± 2.65^b^
Met	93.67 ± 2.71^a^	88.54 ± 1.42^a^	90.24 ± 2.32^a^	88.19 ± 1.88^a^	88.94 ± 1.48^a^	77.34 ± 2.10^b^	75.72 ± 2.12^b^
Phe	93.53 ± 2.07^a^	87.79 ± 2.33^a^	92.12 ± 2.95^a^	87.62 ± 2.18^a^	93.12 ± 2.22^a^	77.73 ± 1.59^b^	73.35 ± 2.33^b^
Thr	91.36 ± 2.19^a^	88.71 ± 3.09^a^	87.21 ± 2.12^a^	85.77 ± 1.79^a^	90.65 ± 2.01^a^	76.12 ± 2.83^b^	74.70 ± 2.04^b^
Val	93.40 ± 1.95^a^	88.34 ± 1.45^a^	87.58 ± 2.15^a^	87.28 ± 2.21^a^	92.47 ± 1.53^a^	78.23 ± 2.13^b^	75.28 ± 2.68^b^
Nonessential amino acids							
Cys	88.24 ± 2.65^a^	86.45 ± 2.10^a^	87.91 ± 2.33^a^	88.08 ± 2.47^a^	87.49 ± 2.17^a^	76.27 ± 1.24^b^	74.22 ± 2.13^b^
Tyr	90.22 ± 2.10^a^	87.18 ± 1.84^a^	87.99 ± 2.80^a^	85.18 ± 1.41^a^	91.17 ± 2.92^a^	77.51 ± 2.18^b^	73.19 ± 2.87^b^
Ala	91.84 ± 1.36^a^	87.43 ± 2.12^a^	91.92 ± 2.20^a^	87.11 ± 2.54^a^	89.71 ± 2.95^a^	74.54 ± 2.62^b^	73.15 ± 2.30^b^
Asp	92.28 ± 1.86^a^	87.29 ± 2.29^a^	89.16 ± 2.82^a^	85.91 ± 1.68^a^	90.41 ± 2.27^a^	77.31 ± 2.29^b^	75.51 ± 3.09^b^
Glu	87.70 ± 1.72^a^	86.41 ± 2.77^a^	86.48 ± 2.09^a^	85.26 ± 2.27^a^	88.76 ± 3.23^a^	76.43 ± 2.60^b^	72.37 ± 2.82^b^
Gly	91.06 ± 2.33^a^	90.30 ± 1.97^a^	89.33 ± 1.55^a^	87.16 ± 2.15^a^	90.11 ± 2.16^a^	75.22 ± 2.25^b^	74.31 ± 2.83^b^
Pro	93.65 ± 1.98^a^	90.79 ± 2.41^a^	92.46 ± 2.88^a^	88.30 ± 1.72^a^	92.31 ± 2.96^a^	77.13 ± 2.87^b^	75.45 ± 3.55^b^
Ser	92.61 ± 2.18^a^	89.33 ± 2.69^a^	90.32 ± 2.54^a^	89.85 ± 2.30^a^	91.53 ± 2.67^a^	76.26 ± 2.90^b^	76.97 ± 2.22^b^

*Note*: Means ± SD (*n* = 3) with the same superscript small letter in the same row are not significantly different (*p* > 0.05). Tryptophan could not be analyzed because of its destruction during acid hydrolysis.

Abbreviations: AKM, Antarctic krill meal; HFM, hydrolyzed feather meal; MBM, meat and bone meal; NFM, native fish meal; NSM, native shrimp meal; PBPM, poultry by-product meal; PFM, Peruvian fish meal.

## Data Availability

The data that support the findings of this study are available from the corresponding author upon reasonable request.
